# Enriched Catalytic Activity of TiO_2_ Nanoparticles Supported by Activated Carbon for Noxious Pollutant Elimination

**DOI:** 10.3390/nano11112808

**Published:** 2021-10-22

**Authors:** Suriyaprabha Rajendran, Gajendra Kumar Inwati, Virendra Kumar Yadav, Nisha Choudhary, Mitesh B. Solanki, Magda H. Abdellattif, Krishna Kumar Yadav, Neha Gupta, Saiful Islam, Byong-Hun Jeon

**Affiliations:** 1 School of Nanosciences, Central University of Gujarat, Gandhinagar 302030, Gujarat, India; sooriyarajendran@gmail.com (S.R.); nishanaseer03@gmail.com (N.C.); 2 Department of Chemistry, D. P. Chaturvedi College, Rani Durgavati University, Seoni, Jabalpur 480661, Madhya Pradesh, India; gajendrainwati@gmail.com; 3 Department of Microbiology, School of Sciences, P P Savani University, Kosamba 394125, Gujarat, India; yadava94@gmail.com; 4 Step-Up Jewels PVT. Ltd. Khatodara Gate, Surat 395002, Gujarat, India; mitesh4physics@gmail.com; 5 Department of Chemistry, College of Science, Taif University, P.O. Box 11099, Taif 21944, Saudi Arabia; m.hasan@tu.edu.sa; 6 Faculty of Science and Technology, Madhyanchal Professional University, Ratibad, Bhopal 462044, Madhya Pradesh, India; envirokrishna@gmail.com; 7 Institute of Environment and Development Studies, Bundelkhand University, Jhansi 284128, Uttar Pradesh, India; nhgupta83@gmail.com; 8 Civil Engineering Department, College of Engineering, King Khalid University, Abha 61413, Saudi Arabia; sfakrul@kku.edu.sa; 9 Department of Earth Resources and Environmental Engineering, Hanyang University, Seoul 04763, Korea

**Keywords:** nanocatalysts, activated carbon, effluent, titania, anatase

## Abstract

Cleaning wastewater has become one of the most serious issues for a number of scientists and researchers in recent years, as water is the most basic need for the daily life of humans. There has been a focus on the removal of noxious pollutants from wastewater effluents by using nanocatalysts owing to their unique physicochemical actions and stability. Herein we manufactured TiO_2_ nanoparticles supported by activated carbon (AC-TiO_2_) using a cost-effective sonochemical method. The band structures of the AC-TiO_2_ and TiO_2_ were modified from 3.2 to 3.1 eV, thus increasing the catalytic activity. The structural, optical and anatase crystal phase properties, with morphological confirmation, were studied by applying UV-DRS, PL, FESEM, XRD, along with HRTEM, respectively. The specific surface area, calculated by BET analysis, was found to be ~241 m^2^/gm and ~46 m^2^/gm for AC-TiO_2_ and TiO_2_. The degradation efficiency of the as-prepared nanocatalysts against the very toxic but rarely studied organic textile dye pollutant RO 84 was investigated and 97% efficiency were found for the AC-TiO_2_ as compared to pure TiO_2_, which is a highly appreciated finding in the catalytic dye degradation application domain. Such surface-modified nanocatalysts could be further implemented for the treatment of wastewaters/waste effluents released from chemical industries, laboratories and other sources.

## 1. Introduction

Organic or inorganic molecule-based contaminants present in wastewater have been found with change into various chemical states, compositional ratios or other heterocyclic structures such as aniline intermediate species [1,2,3]. Dye components and their derivatives affect the health of the environment by contaminating water, soil, and air. Thus, there has been a motivation for researchers to design effective technologies to resolve such environmental issues, especially in the dye and textile industries. From this point of view, dye fabrication, textile research, and production plants are now required to treat their effluents for the purpose of ensuring the safety of human life and an ecofriendly environment. A number of studies have been carried out in academic and industrial sectors to minimize the high load of organic and solid contents of discharged wastewaters [4,5], which mainly consist of biological toxic, organic, and inorganic impurities which threaten water scarcity for both humans and the ecosystem. In catalysis applications, the decomposition of effluents/contaminants from wastewater using photocatalytic processes are appreciated by the dye and textile sectors. Photochemical reactions using nanocatalysts and technologies have become the simplest route to treat the organic (heterocyclic/aromatic)-based wastes and toxic substances. Doping of metal oxides and their hybrid structures with suitable metals, ions, or atomic layered structures is applied to improve the catalytic performance. Such modified structures are widely explored due to the efficient redox reactions with higher electrons and hole recombination rates. The formation of reactive species such as transient hydroxyls (•OH) and holes often regulate the catalytic process owing to the delayed recombination of free charges [4,6]. Fundamentally, photodegradation can be understood to depend on two classes of catalysts, homogeneous and heterogeneous. Homogeneous processes are often conducted using continuous UV light irradiation in conjunction with specific oxidants, e.g., ozone and hydrogen peroxides. In the case of heterogeneous catalysis, oxidants in conjunction with crystalline photoactive catalysts are required to trigger the catalytic mechanism. Light-based degradation effects depend on different reaction parameters, including the reaction medium (pH), amount of catalyst and the nature of the targeted chemical contaminants or organic effluents [7,8]. By following a chemical treatment approach various pollutants, especially dye contents, pathogens and toxic heavy metals, could be mineralized and therefore the photocatalysis chemistry is promoted by using appropriate chemicals (catalysts). Photocatalytic reactions can take place under certain experimental parameters which felicitate a feasible approach to regulate the catalytic actions and appreciated for wastewater cleaning and hence solar radiation or UV irradiation may be employed as a light source to activate the catalyst [8]. Among the various catalysts, TiO_2_ nanoparticles are semiconductor metal oxide photocatalysts that promise to offer an inexpensive, desirable method for wastewater purification [4,8]. It has been mentioned that TiO_2_ shows stronger catalytic action under a certain light intensity when in the form of anatase, while other phases like rutile exhibit a comparably weak photoactive catalytic activity and hence the anatase form of TiO_2_ is more appreciated. Systems to improve the photoactivity of TiO_2_ have been formulated following several preparation procedures to control the shape and surface modification of anatase phase titania [9,10,11]. The pairing of electrons-holes could be considered a limiting factor for TiO_2_ semiconducting nanocatalysts. Therefore, various efforts have been undertaken to make TiO_2_ a more successful photocatalyst by doping it with different additives such as metal oxides or carbon composites. The altered structures of the nanodimensional hybrid substances open multidirectional options due to the larger surface and adjustable morphological properties [12,13,14]. Optoelectronic and photoelectrochemical processes under light modulation could be regulated by inserting multivalent ions at a fixed stoichiometric concentration [15,16]. Metamaterials can also be constructed in order to achieve desirable properties by introducing suitable chemical constituents or elements. This represents an advanced technique with wider uses in material modification [2,11]. Carbon-containing compounds can be classified into different types according to their functionalities, such as 2D graphene, its reduced derivatives, carbon nanotubes (CNTs) or fiber-like shapes including activated carbon [2,17]. Among these, activated carbons are highly functionalized carbon allotropes that can be derived from any natural carbon source. These activated carbons have a greater tendency to display enhanced adsorption properties due to their porosity and active surface area. The changes in elemental ions in TiO_2_ with structural tuning represent an outstanding research area in the photocatalysis sector [17,18]. Particularly, the bandwidth of the TiO_2_ could be changed by the adding elements such as C, Br and others which changes the band position of the conduction and valance bands allowing single or multivalent electron transformations [19]. The optimized band values allow a discrete level of light into the semiconducting materials like TiO_2_, especially in catalytic systems. In the semiconducting nanomaterial or metamaterials, the band structures and physicochemical aspects are modulated by appropriate experimental schemes to ensure efficient catalytic activity [20,21]. Non-metal elements, mainly tetravalent carbon allotropes, can be used to boost the physical and chemical properties of TiO_2_ for the mineralization and elemental degradation of hazardous molecules [22]. That is why heterogeneous catalysts have been highly studied for the field of degradation for their environment impact in the dye and textile sectors [22,23]. In contrast, different classes of metal-oxide compositions are employed for the decomposition of various types of dye and the purification of water by removing heavy elements/compounds [15,21]. Generally, electromagnetic intensity responds to the catalyst at a certain power density or energy whereas the TiO_2_ responds to UV light in the range under 370–415 nm. However, the visible range of the electromagnetic spectra can also be used to induce specific photocatalysis processes by manipulating other catalytic factors such as bandgap energy, catalyst morphologies and the elemental constituents. The photocatalytic decompositions of dyes using TiO_2_ can be classified on the basis of the redox reactions taking place, the chemical composition and the nature of co-product(s) which could be explained as follows: first, photo decolorization involves chemically active redox process in terms of photo-oxidation or reduction, resulting in initial chemical phases based on reduction or oxidation redox mechanisms [24,25]. Second, light-treated dye components, which means non-reactive co-products, are produced by photodegradation approaches. This has been a highly preferred term for the catalytic chemistry on dye molecules and their derivatives. Lastly, the total breakdown of the dye components into possible preferred chemical species including water, nitrogen-containing gases, and this process is called photomineralization [24,26,27]. In this work, a high photocatalytic active TiO_2_ (anatase) was modified in terms of band position by doping with active carbon (AC-TiO_2_) NPs and used to degrade the rarely studied organic dye molecule RO84. The structures with altered structural and surface properties were then confirmed as an efficient photocatalyst to remove pollutants, especially for the degradation of RO8 in industrial wastewaters. These structural and surface studies of the pure and supported TiO_2_ are focused on the possible environmental impact.

## 2. Materials and Methods

All reagents were of analytical grade. Titanium isopropoxide, was purchased from Sigma Aldrich (Taufkirchen, Germany), while acetic acid (SRL, Ahemdabad, India), nitric acid (SRL, Ahemdabad, India, 69%) and ethanol (Schinzen, China) and activated carbon (HiMedia, New Delhi, India) were also used.

### 2.1. Preparation of Titanium Oxide Powder

Titanium isopropoxide was the selected starting material. A 1 M solution in ethanol (50 mL) was prepared. About 30 mL of this as-prepared precursor solution was taken and acetic acid (0.1 M) and nitric acid (0.1 M) were added dropwise under constant stirring to moderate the chemical reaction under ultra-sonication at a fixed frequency of 40 kHz. The reaction was carried out for 4 h at 70 °C at an open atmosphere. The white cake formed was separated and dried under vacuum for a night at 25 °C. Then the obtained solid powder was calcined at 700 °C for complete phase transformation.

### 2.2. TiO_2_ Supported on Activated Carbon

The carbon source, namely activated carbon, was purchased in the form of pellets which were crushed manually and then mixed with distilled water. The carbon suspension was added into the precursor Ti [OCH (CH_3_)_2_]_4_ which was further reduced by acetic acid. The grey colored product was further dried and calcined to obtain the TiO_2_ NPs. The obtained activated carbon TiO_2_ (C/TiO_2_) was further characterized.

### 2.3. Characterization Techniques

The techniques used for the characterization were ultraviolet (UV) spectroscopy for the optical properties and bandgap studies, X-ray diffraction (XRD) for the structural phases, the structural morphology and elemental composition was performed using a field emission scanning electron microscope (FESEM) and high-resolution transmission electron microscopy (HR-TEM), respectively, while the characteristic vibrational modes for the expected functional groups were identified using Fourier-transform infrared spectroscopy (FTIR). Brunner-Emmett-Teller (BET), photoluminescence (PL) was used for surface area and luminescent property measurements.

### 2.4. Photocatalytic Experiments

The photocatalytic studies of the synthesized catalysts against the organic textile dye RO84 were carried under a mercury lamp using visible light. Firstly, 5, 10 and 15 ppm stock solutions of RO8 dye were prepared and then 0.1 to 0.3 g of photocatalyst was mixed with 20 mL of the stock solutions separately. The catalytic activity was determined by turning on the mercury lamp and the light-treated material was sampled at 10 min intervals. The treated samples were centrifuged and then their UV-Vis spectra were recorded to follow the degradation. The degradation % of the RO84 was calculated by applying the following equation:

$$\mathrm{Photodegradation}\;\mathrm{efficiency}=\left(1-\frac{C}{C_{0}}\right)\times 100$$

The rate constant *(k*) was calculated by finding the first-order kinetics of the pure and AC-TiO_2_. The formula used for the kinetic rate is given below:

$$In\left(\frac{C}{C_{0}}\right)=-kt$$

where *C*_0_ is the initial concentration of the RO8 dye solution, and *C* is the concentration of dye after light exposure for a certain time (*t*).

## 3. Results and Discussion

### 3.1. XRD Spectrometry: Phase Confirmations

**Figure 1:**
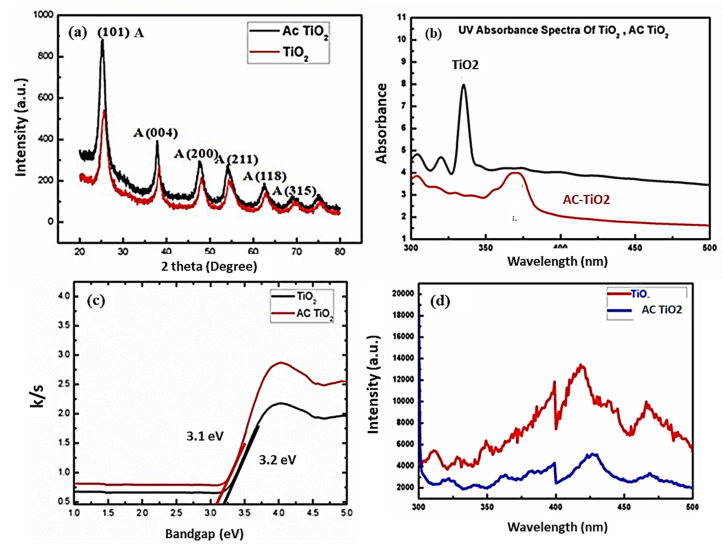
(**a**) XRD profile for unsupported TiO_2_ and AC-TiO_2_, (**b**) optical absorption (UV-Vis) curve for TiO_2_ and AC-TiO_2_, (**c**) UV-Visible study (DRS) of TiO_2_ and AC-TiO_2_ and (**d**) photoluminescence response for pure TiO_2_ and AC-TiO_2_ structure.

The unsupported and carbon-based TiO_2_ NPs were analyzed in order to confirm their crystalline structure. The crystalline profiles of TiO_2_ and doped samples were recorded using XRD and are depicted in Figure 1a. The main peaks at the positions of 25.3°, 37.8°, 48.0°, 53.8°, 55.1°, and 62.8° indicate formation of TiO_2_ anatase with respect to the 101, 004, 200, 211, 118 and 315 crystallographic planes (JCPDS number 75-15437) [26,28]. The XRD results thus confirm that an anatase crystalline phase of TiO_2_ is formed after the heat treatment at 500 °C in an open atmosphere environment. The diffraction peak intensity increased in the case of AC-TiO_2_ nanocomposites after adding the AC in a certain amount. Remarkably, no further peaks were observed in the AC-TiO_2_ composites at the 24.6° location, which is assigned to the loaded AC showing diffracted peaks corresponding to the (002) crystalline plane. This could be because this characteristic peak of AC would be masked by the anatase crystal faces of TiO_2_ located at 25.3°. Furthermore, the crystalline sizes of the formed TiO_2_ photocatalyst could be estimated using Scherer’s formula, especially for the characteristic diffraction peak and crystallographic planes and a range of 26.9 and 29.3 nm (crystalline sizes) for pure and AC-TiO_2_ was observed [26,29].

### 3.2. UV-Absorbance Spectra

Figure 1b displays the UV optical absorbance for TiO_2_ and AC-TiO_2_ NPs, displaying a prominent absorption hump at around λ < 400 nm. The main optical absorption features were found at around 200–400 nm, and are due to the electron transitions from the valence to the conduction band of the TiO_2_. These optical ranges confirmed the formation of the TiO_2_ in anatase form.

TiO_2_ surrounded by carbon layered structures absorbs a higher range of light below the visible zone of the electromagnetic spectrum (≈400 nm). Basically, the spectral intensities shift for the AC-TiO_2_ as near to the TiO_2_ energy zone [29,30]. The carbon often allows Ti to sustain the trivalent state along with the O^2^ vacancy state, depending upon the conduction and valence band positions. Activated C compounds present a larger absorption of the visible spectral range, confirmed by the change in grey color [2,31]. There was a significant difference reported for the light absorption capacity for the pure and carbon-loaded TiO_2_. Here the bandgap modification concept was introduced to degrade the particular RO84 organic dye molecules. The modified band gap energies (3.1 and 3.2 eV) for pure and AC-TiO_2_ are not often studied for organic textile dye effluents, thus, the obtained band position responds actively for the catalytic destruction of RO84 dye.

### 3.3. UV-DRS Spectra

UV-DRS results for the powdered AC-TiO_2_ and TiO_2_ are depicted in Figure 1c. The absorption peak at around 400–450 nm was assigned to the AC-TiO_2_ along with the TiO_2_. The resulting spectra revealed that the optimized experimental route is practical for producing the anatase state with the aim of absorbing visible light in the electromagnetic spectrum. Bandgaps values for both samples were determined, and found to be 3.1 and 3.2 eV, corresponding to the doped AC-TiO_2_ and TiO_2_ nanopowders, respectively [2,32]. Notably, the bandgap of AC-TiO_2_ was found to be less than that of the pure TiO_2_ which could be due to the interference of the activated carbon. This modified AC-TiO_2_ band was found to be highly desirable for the catalysis of RO84 textile organic dye decomposition, which is a new finding for particular wastewater treatment applications.

### 3.4. Photoluminescence Study

A photoluminescence study was done for all the samples under normal conditions (RT) and the emission peak response is displayed in Figure 1d. The anatase phase of the respective materials emits light in the green luminescent band and therefore the study has been carried out to confirm the crystal phase. The luminescence process mainly occurs due to the electron transitions from the trapping levels of conduction bands to the valance band. Certain intensities of the light allow the transit of free electrons, resulting in emission bands (luminescent bands). [32]. There is a significant change in the peak intensity of the unsupported and supported TiO_2_ because of the doped carbon in certain experimental setups. Thus, the used method was found to be very appropriate for the synthesis of the anatase phase in both the samples [31,32].

### 3.5. BET Analysis: Adsorption Spectra

**Figure 2:**
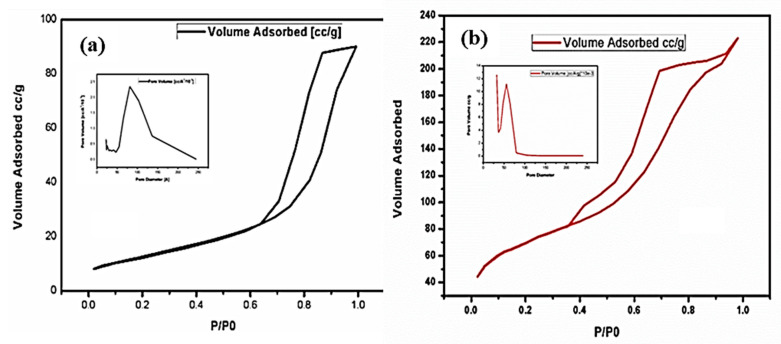
BET absorption spectra of (**a**) unsupported TiO_2_ and (**b**) supported AC/TiO_2_.

Figure 2 illustrates the N_2_ adsorption with the desorption isotherm, distribution of pore sizes of anataseTiO_2_ and AC-TiO_2_, respectively (Figure 2a,b). Type-II isotherm character was shown by the TiO_2_, whereas a type-IV isotherm is demonstrated for the AC-TiO_2_ samples in this scenario, Type-II could be confirmed based on the disappearance of hysteresis in the obtained graph and a valid difference was noticed between the adsorption and desorption areas for TiO_2_ samples above/Po > 0.6 pressure. The occurrence of the gap revealed that larger pores are available on the surfaces with voids. The closed adsorption-desorption curve indicated by its narrow size mesoporous (2–50 nm as per IUPAC) structures on the surface at P/Po ~ 0.4 relative pressure. Here the hysteresis nature is assigned to the capillary condensation character for both structures considering mesoporous and micropores. The calculated specific surface area was ~241 m^2^/gm for AC-TiO_2_ solid powder which was larger than that of the TiO_2_ powder (~46 m^2^/gm), so the change in the surface area also supports the modification conditions applied during the surface alterations and morphological changes caused by doping [25,27].

### 3.6. Morphological Studies: HRTEM Analysis

**Figure 3:**
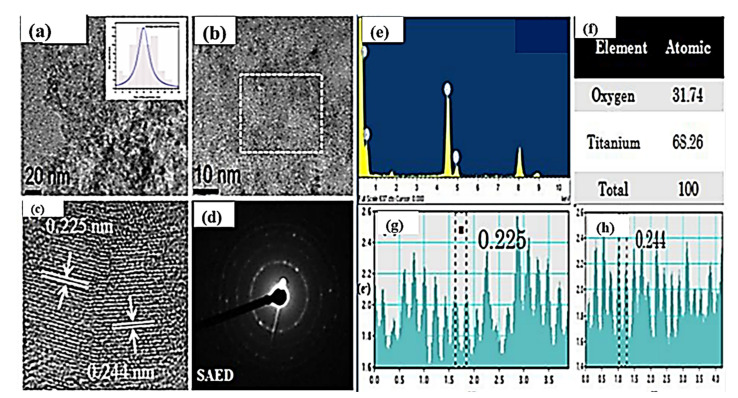
HRTEM images of synthesized TiO_2_ NPs, (**a**) TEM micrographs with histogram, (**b**) HRTEM images, (**c**) lattice parameter and (**d**) SAED pattern of TiO_2_. (**e**) EDX spectra (**f**) atomic % of Ti and O, (**g**,**h**) particle d-spacing analysis.

Figure 3a–h present the HRTEM images of pure TiO_2_ NPs after the calcination at 500 °C. The average particle size was found to be 24.5 nm and the interplanar distance (d) was observed as 0.244 nm with crystalline behaviour, evidenced by the SAED pattern. Furthermore, the prepared powdered structures of the samples were assigned to the Ti and O atomic %, respectively.

**Figure 4:**
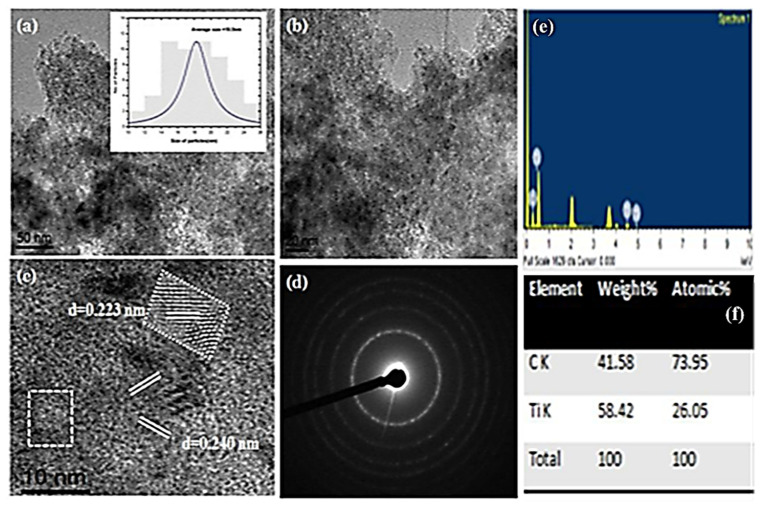
HRTEM and EDX images of synthesized AC-TiO_2_ NPs, Image (**a**,**b**) are TEM images, with histogram, lattice parameter (**c**,**d**) SAED pattern of AC-TiO_2_, (**e**,**f**) shows the EDX image with atomic percentages.

Figure 4a–f show HRTEM and EDX images of the AC-TiO_2_. Micrographs of the analysed samples show powdered carbon substances surrounding the open face of TiO_2_.

The average size displayed by the histogram is found to be 25.4 nm and crystalline behaviour was estimated by the analysing the SAED which shows a higher solid-state for the AC- supported TiO_2_. The obtained values for the pure and supported TiO_2_ particles were found to be smaller than the XRD sizes as refers to crystallite sizes. Figure 4e,f confirmed the elemental compositions for the doped carbon along with the Ti percentage, studied by EDX peak analysis in terms of atomic weight %.

## 4. Removal of Organic Pollutants

The characteristics of the band positions, opacity and the surface of the anatase form of TiO_2_ nanostructures revealed acceptable potentials for photocatalytic activity. Among all three crystalline states, anatase is widely adopted along with hybrid structures doped with lightweight atoms including anions (halogens) because of their liquid synthesis procedures [33,34]. The doping has been studied and extensively explored towards catalytic applications of TiO_2_ semiconductors [35,36,37,38]. In this work, we manufactured TiO_2_ and carbon-supported TiO_2_ and characterized them. The degradation of the textile dye molecule RO84 by the as-designed photocatalyst was further studied as a new approach to dye degradation studies. The as-modified band structure was influenced by visible range of electromagnetic radiation, hence demonstrating catalytic activity against the RO84 organic structure.

### Photocatalytic Degradation of RO84

**Figure 5:**
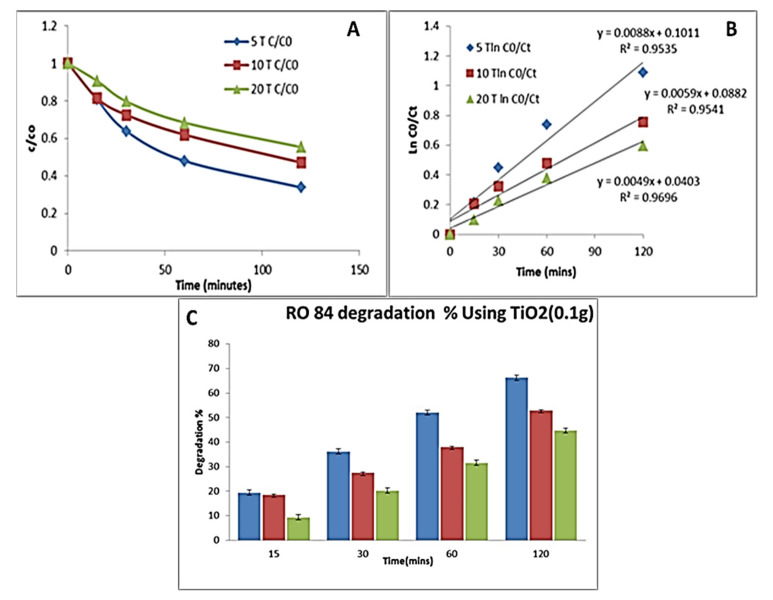
(**A**,**B**) Kinetic degradation plot of RO84 with TiO_2_ (0.1 g). (**C**) Degradation % of RO84 using TiO_2_ (0.1 g).

Initially, 0.1 g of sample was used, which was then varied up to 0.3 g to optimize the degradation performance (as detailed in the Materials and Methods section). Five, 10, and 20 ppm concentration of the RO84 was taken and firstly reacted with 0.1 g of catalysts (TiO_2_ and ACTiO_2_). The TiO_2_ shown 66% degradation at a low concentration of dye, determined by evaluating the first-order reaction rate as shown in the kinetics graphs (Figure 5A,B).

**Figure 6:**
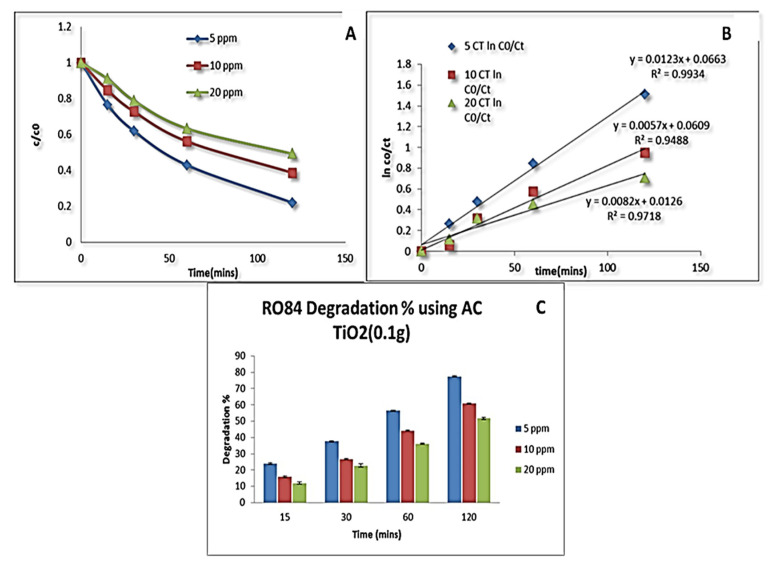
(**A**,**B**) Degradation kinetics of RO84 with AC-TiO_2_ (0.1 g). (**C**) Degradation percentage of RO84 using AC-TiO_2_ (0.1 g).

A 44% degradation was found at a higher concentration for the same amount of catalyst. The kinetic plot was obtained for the TiO_2_ (0.1 g) followed by first-order fitting. The rate constants were 0.008, 0.0059 and 0.0049 min^−1^ with respect to 5, 10, 20 ppm of dye with R^2^ equal to 0.95. The degradation kinetics curves with respect to time (min) are shown in Figure 5C. However, when the same concentration of the dye was used for the AC-TiO_2_ nanocrystals to check the degradation rate, it was found to be 78% and 51% for the lower and higher concentrations, respectively. The k values were found on the order of 0.01, 0.005, and 0.008 min^−1^ for the used concentration of RO84 based on first order fits. Figure 6A–C display the kinetic studies of the degradation % vs. time of RO84 using 0.1 g of AC-TiO_2_. AC with 10–20 nm sizes was decorated on a TiO_2_ surface by Xing et al., [39] using a sol-gel technique and around 93% degradation of RhB was observed using UV light. They proposed a dual mechanism for the absorption and degradation activity for the removal of RhB in water. The adsorption efficiency was increased by the availability of the open surfaces mainly due to the activated carbon on the TiO_2_ structure.

Nearly two-fold better catalytic efficiency was reported by Amran and co-authors [40] by using a modified TiO_2_ surface. Carbon was used to alter the bandgap of TiO_2_ which was reduced to 2.38 eV and employed to enhance the performance in the visible light range. The methylene blue molecule was degraded using this AC-doped TiO_2_ and 82.67% efficiency was recorded due to a larger absorption of visible light by carbon.

To minimize the loss of designed composites (AC/TiO_2_) the polymer fiber was immobilized and that was the reason for the better recyclability performance during the catalytic measurements.

**Figure 7:**
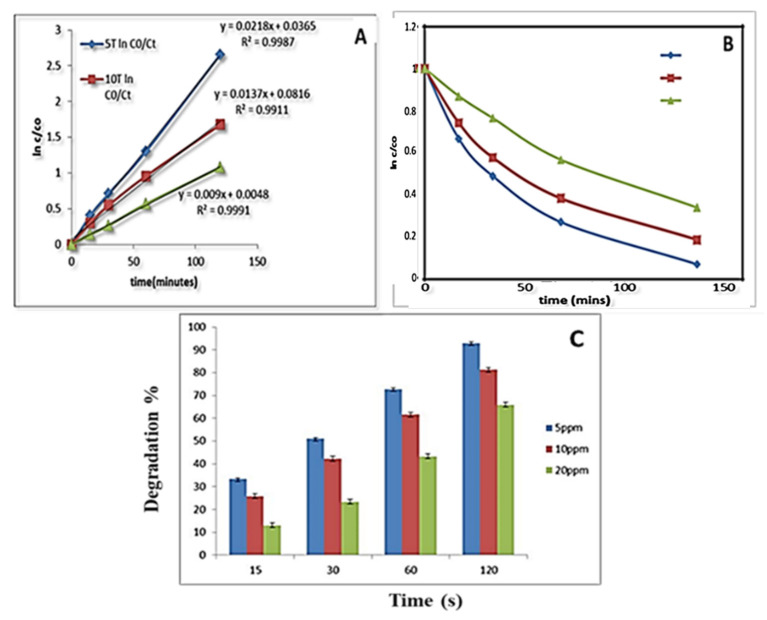
(**A**,**B**) RO84 degradation kinetics using TiO_2_ at 0.3 g. (**C**) Degradation % of RO84 using TiO_2_ (0.3).

A certain amount of catalyst was found to be much more effective in the degradation of the RO84 dye as we changed the amount from 0.1–0.3 g. The experimental conditions also played a key factor to enhance the reaction rate and thus the route employed is found to be very useful for this particular catalyst (TiO_2_, and AC-TiO_2_). By increasing the concentration of catalyst (TiO_2_) to 0.3 g a degradation of up to 92% was achieved for a lower concentration of dye in which the reaction follows first-order kinetics. The R^2^ values are above 0.95 and the k values are found to be 0.02, 0.01 and 0.009 min^−1^ for 5, 10, 20 ppm of RO84, respectively, as depicted in Figure 7A–C. The degradation percentage was found to be 65% at a higher concentration of dye. Significantly, the kinetic rate of the photocatalysis is regulated by the recombination time of electron-holes of the semiconductors. TiO_2_ has already been implemented as one of the finest catalysts for degradation of contaminants due to its characteristic band structures and crystal phases [41,42]. In our study it has been modified structurally and applied to achieve an enhanced functionality for the decomposition of RO84.

**Figure 8:**
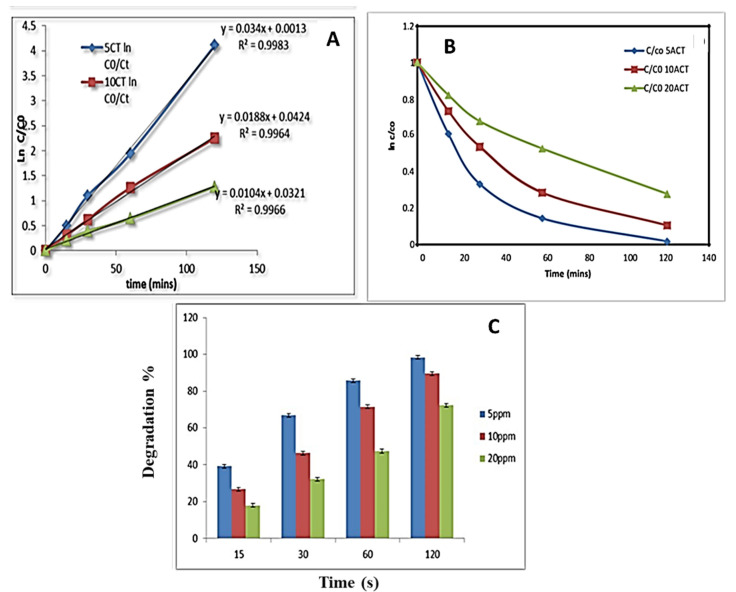
(**A**,**B**) RO84 degradation kinetics using AC-TiO_2_ at 0.3 g, (**C**) Degradation % of RO84 using AC-TiO_2_ (0.3 g).

The larger amount of AC-TiO_2_ (0.3 g) induces a higher degradation rate of RO84 and the corresponding kinetic plots are shown in Figure 8. The kinetic rate could be determined in the form of k values, found as 0.034, 0.01 and 0.010 min^−1^ for 5, 10, 20 ppm of RO84, respectively. A 97% degradation efficiency was found for the lower concentration of taken dye using a 0.3 g amount of photocatalyst, as shown in Figure 8A–C, whereas 72% decomposition efficiency was obtained for a higher concentration of the dye for the same catalyst. Therefore, the activated carbon provided a larger area to adsorb RO84 molecules with strong binding onto the designed catalyst surface [42,43]. The bandgap engineering of anatase TiO_2_ supported by AC also provides a feasible environment to degrade the pollutants in our case. The crystalline and stabilized AC/TiO_2_ was thus found to be very effective based on the catalysis improvement caused by the bigger surface and the controlled structural properties. The adsorption and light absorption at a certain light intensity was modulated by the morphological changes in the TiO_2_ and therefore it has been expressed as an enriched catalytic activity for RO84.

## 5. Conclusions

A crystalline form of TiO_2_ anatase supported by activated carbon was prepared. Its structural, optical, and luminescence properties were confirmed. Narrow-sized AC-TiO_2_ and TiO_2_ NPs were observed by the use of XRD and HRTEM, whereas the surface area was studied by BET experiments. The surface areas for TiO_2_ and AC-TiO_2_ were found to be ~241 m^2^/gm and ~46 m^2^/gm, respectively. The atomic percentage elemental compositions of pure TiO_2_ and AC-TiO_2_ were also determined by an EDX study. An enhanced degradation rate of the pollutant RO84 was obtained using TiO_2_ and AC-TiO_2_ nanocatalysts which showed a maximum of 92% and 97% efficiency with the optimized amount (0.3 g) of photocatalyst, respectively. The activated carbon possesses a larger active area to facilitate the outstanding catalytic action of the TiO_2_ owing to its chemical stability with unique physicochemical functionalities. The modified bandgap of the as-prepared photocatalyst was found to be highly desirable to decompose the molecular structure of the target RO84, which is a major finding in wastewater remediation. These modified nanostructures could be further applied to multiple species in environmental applications.
